# Взаимосвязь конституционально-экзогенного ожирения и гормонально-микроэлементного статуса у мальчиков-подростков с задержкой полового развития

**DOI:** 10.14341/probl13155

**Published:** 2024-03-06

**Authors:** Н. А. Друккер, Н. В. Палиева, Ю. А. Петров, В. А. Попова

**Affiliations:** Ростовский государственный медицинский университет; Ростовский государственный медицинский университет; Ростовский государственный медицинский университет; Ростовский государственный медицинский университет

**Keywords:** лептин, токсические химические элементы, эссенциальные химические элементы, мальчики-подростки, пубертат, задержка полового развития, детское ожирение

## Abstract

ОБОСНОВАНИЕ. Детское ожирение и темпы его распространения — серьезная угроза репродуктивному здоровью нации, особенно среди мальчиков, является фоном для задержки полового развития и в дальнейшем приводит к нарушению фертильности.ЦЕЛЬ. Изучение особенностей соотношения уровня лептина и ряда токсических и эссенциальных химических микроэлементов в биологических средах у мальчиков-подростков 13–14 лет с ожирением и задержкой полового развития.МАТЕРИАЛЫ И МЕТОДЫ. Исследованы и сформированы три группы подростков 13–14 лет: основная — с конституционально-экзогенным ожирением 1–2 степени (1 — 20 мальчиков без вторичных признаков полового созревания; 2 — 24 мальчика с 2–4 стадией полового созревания по Таннеру) и сравнения (3 — 15 мальчиков с нормальной массой тела и без отклонений в половом созревании). Определялись в утренней моче уровень свинца, цинка, селена, хрома и марганца абсорбционным методом; в сыворотке крови — лептина методом иммуноферментного анализа. Статистический анализ данных проводили в среде MeoCape 11.4.2 Statistica, непараметрический корреляционный анализ — по Спирмену и расчет t-критерия Стьюдента для независимых выборок, достоверность результатов при р< 0,05.РЕЗУЛЬТАТЫ. Установлено, что для подростков с ожирением характерен определенный сдвиг в содержании токсических и эссенциальных микроэлементов, вектор которого смещен в сторону преобладания уровней токсических химических элементов, в частности свинца, и снижение эссенциальных элементов, таких как цинк, селен, хром и марганец. Однако более выраженное смещение значений в дисбалансе микроэлементов уже нарушает не только обменные процессы в организме детей-подростков, но и приводит к нарушению пубертата — к задержке полового развития.ЗАКЛЮЧЕНИЕ. В организме мальчиков-подростков с ожирением и задержкой полового развития прогрессируют процессы окислительного стресса, тканевой гипоксии на фоне избытка лептина, накопления тяжелых металлов и дефицита эссенциальных микроэлементов. Менее выраженные сдвиги в содержании лептина и микроэлементов у мальчиков-подростков определяют сбой в нейроэндокринной регуляции, но не затрагивают уровень полового созревания. Гомеостаз гормонально-микроэлементного состава обеспечивает гармоничное развитие мальчиков-подростков.

## Abstract (en)

BACKGROUND: Childhood obesity and the rate of its spread is a serious threat to the reproductive health of the nation, especially among boys, being a background for delaying sexual development and further disrupting fertility.AIM: To study the peculiarities of the ratio of the level of leptin and a number of toxic and essential chemical trace elements in biological environments in adolescent boys aged 13–14 years with obesity and delayed sexual development.MATERIALS AND METHODS: Three groups of adolescents aged 13–14 years were studied and formed: the main ones — with constitutional exogenous obesity of 1–2 degrees (1–20 boys without secondary signs of puberty; 2 — 24 boys with 2–4 stages of puberty according to Tanner) and comparisons (3 — 15 boys with normal body weight and without deviations in puberty). The level of lead, zinc, selenium, chromium and manganese in the morning urine was determined by the absorption method; in the blood serum — leptin, by the method of enzyme immunoassay. Statistical analysis of the data was carried out in the MeoCape 11.4.2 Statistica environment, nonparametric Spearman correlation analysis and calculation of the Student’s t-test for independent samples, the reliability of the results at p< 0.05.RESULTS: It was found that adolescents with obesity are characterized by a certain shift in the content of toxic and essential trace elements, the vector of which is shifted towards the predominance of levels of toxic chemical elements, in particular, ead, and a decrease in essential toxic elements, such as zinc, selenium, chromium and manganese. However, a more pronounced shift in the values in the imbalance of trace elements already violates not only the metabolic processes in the body of adolescent children, but also leads to a violation of puberty - to a delay in sexual development. CONCLUSION: In the body of adolescent boys with obesity and delayed sexual development, the processes of oxidative stress, tissue hypoxia are progressing against the background of excess leptin, accumulation of heavy metals and deficiency of essential trace elements. Less pronounced shifts in the content of leptin and trace elements in adolescent boys are determined by a failure in neuroendocrine regulation, but does not affect the level of puberty. The homeostasis of the hormonal-microelement composition ensures the harmonious development of adolescent boys.

## ОБОСНОВАНИЕ

Всемирная организация здравоохранения (ВОЗ) объявила, что 15 млн детей и подростков Европейского региона планеты имеют ожирение, и к 2025 г. прогнозируется повышение этого показателя до 70 млн в группе детей до 5 лет [1]. Полагают, данная ситуация — результат технического прогресса и индустриализации, которые повлияли на биологический облик современного человека, отразившись не только на соматическом здоровье нынешнего поколения, но и на репродуктивном благополучии [2]. Так как замечено, что параллельно с ростом детского ожирения в последние десятилетия стал наблюдаться рост случаев нарушения пубертата и значительный рост бесплодия, в частности мужского фактора, чего ранее не отмечалось [3]. Одной из причин увеличения доли мужского бесплодия в общей структуре факторов инфертильности называют задержку полового развития (ЗПР) на фоне детского ожирения. Научные данные свидетельствуют, что у девочек ожирение тоже нарушает пубертат, но, в отличие от мальчиков, приводит к преждевременной активации функции репродуктивной оси [4][5]. Вот почему у мальчиков в 9 раз чаще, чем у девочек, встречается функциональная задержка старта пубертата [6][7].

Принято считать, что в основе этих нарушений лежит десинхронизация деятельности звеньев гипоталамо-гипофизарно-гонадной системы, в реализации функции которой участвует лиганд-рецепторная система кисспептина [8]. Роль посредника между лептином и гонадотропин-рилизинг гормональным секретирующим центром гипоталамуса выполняет кисспептин, регулируя прямую и обратную связь в пределах гонадной оси. Лептин же, не только гормон жировой ткани и модулятор пищевого поведения, но и один из основных гормональных участников биохимического регуляторного каскада пубертата. Известно, что его избыток в юном возрасте тормозит гонадотропную функцию гипоталамо-гипофизарной системы и, соответственно, способствует задержке пубертата [9].

Наряду с нейроэндокринным менеджментом, процессами полового созревания управляют эссенциальные химические элементы (ЭХЭ). Причем дисбаланс последних приводит к сбою нейроэндокринной регуляции репродуктивной системы [10]. Целый ряд исследований, в том числе наши, указывают на определенную взаимозависимость ЭХЭ с группой токсических химических элементов (ТХЭ) и отмечают особый характер соотношения этих ХЭ у мальчиков-подростков с задержкой полового развития (ЗПР) на фоне ожирения. В частности, у них в моче определяется высокий уровень такого ТХЭ, как свинец [11][12]. Показано, что особый тип микроэлементоза может индуцировать возникновение как дефицита, так и избытка массы тела, способствуя целому ряду метаболических осложнений. ТХЭ (свинец, кадмий и др.), по сути, являются физиологическими антагонистами важнейших ЭХЭ (цинк, селен, медь и др.). Определенная палитра ХЭ в организме детерминирует секрецию люлиберинов и гормонов аденогипофиза, что доказывает их значимость в организации работы репродуктивной оси. В ряде территорий, эндемичных по избытку некоторых ТХЭ, присутствие хронического оксидантного стресса в организме может стать дополнительным, чрезвычайно опасным триггером для нарушения здоровья, особенно ребенка в период полового созревания [10][13].

## ЦЕЛЬ ИССЛЕДОВАНИЯ

Изучение особенностей соотношения уровня лептина и ряда токсических и эссенциальных химических микроэлементов в биологических средах у мальчиков-подростков 13–14 лет с ожирением и ЗПР.

## МАТЕРИАЛЫ И МЕТОДЫ

## Место и время проведения исследования

Место проведения. Научно-исследовательский институт акушерства и педиатрии ФГБOУ ВО «Ростовский государственный медицинский университет» Минздрава России, Ростов-на-Дону (НИИАП РостГМУ).

Время исследования. Период с марта 2015-го по сентябрь 2019 гг.

## Изучаемые популяции (одна или несколько)

Популяция: подростки школьного возраста, обследуемые в рамках диспансеризации школьников г. Ростова-на-Дону.

В исследование включены 59 подростков 13–14 лет, из которых сформированы три группы: две основные (1-я и 2-я группы), с конституционально-экзогенным ожирением (КЭО) 1–2 степени и одна — сравнения (3-я группа), с нормальной массой тела. Первая группа — 20 подростков без вторичных признаков полового созревания (объем тестикул менее 3–4 мл); вторая группа — 24 подростка с 2–4 стадией полового созревания по Таннеру и третья группа — 15 мальчиков без отклонений в половом созревании (2–4 стадия полового созревания по Таннеру). В ходе исследования ни один участник не выбыл из группы и не было зарегистрировано ни одного нежелательного явления после инвазивных вмешательств с целью забора биологического материала.

Критерии включения: возраст от 13 до 14 лет; конституционально-экзогенное ожирение (КЭО) 1–2-й степени без вторичных признаков полового созревания (отсутствовал объем тестикул, равный 3–4 мл); с вторичными признаками полового созревания (2–4 стадии по Таннеру) и здоровые мальчики без отклонений в массе тела и половом развитии (2–4 стадии по Таннеру).

Критерии невключения: дети и подростки других возрастов; врожденные и приобретенные пороки развития, включая хромосомные и генетические заболевания; крипторхизм; гипогонадизм; другие эндокринные заболеваниями (сахарный диабет, гипопитуитаризм, гипотиреоз и т.д.).

Критерии прекращения участия в исследовании: отказ от участия в исследовании.

## Способ формирования выборки из изучаемой популяции (или нескольких выборок из нескольких изучаемых популяций)

Сплошной способ формирования выборки.

## Дизайн исследования

Одноцентровое, интервенционное, одномоментное (поперечное), одновыборочное, неконтролируемое, сравнительное исследование.

## Описание медицинского вмешательства (для интервенционных исследований)

С целью получения искомых данных вначале проведена оценка антропометрических показателей и некоторых физиологических особенностей организма подростков, затем проводилось гормонально-микроэлементное исследование биологических сред, полученных в утренние часы (сыворотка периферической крови и моча).

## Методы

Степень полового развития оценивалась согласно стадиям полового развития по Таннеру. Объем тестикул определялся методом УЗИ, ожирение выставлялось на основании расчета индекса массы тела (ИМТ) по формуле Кетле (отношение веса в килограммах к росту в квадрате, выраженному в метрах, кг/м²). Согласно федеральным клиническим рекомендациям «Оценка физического развития детей и подростков» (2017 г.), с учетом рекомендаций ВОЗ (2011 г.), ожирение у детей и подростков от 0 до 19 лет определяли как ИМТ, равный или более +2,0 SDS ИМТ. Степени ожирения оценивались как I — SDS ИМТ 2,0–2,5; II — SDS ИМТ 2,6–3,0; III — SDS ИМТ 3,1–3,9. Критерии невключения устанавливали на основании документального подтверждения возраста (свидетельство о рождении или паспорт), диагнозов в истории болезни ребенка о наличии хромосомных, генетических или других эндокринных заболеваний, подтвержденных результатами клинико-лабораторных и инструментальных исследований, а также общего осмотра (в частности, крипторхизм).

Микроэлементный (МЭ) состав утренней мочи (свинец (Рb), цинк (Zn), селен (Se), хром (Cr) и марганец (Mn)) устанавливался абсорбционным методом, анализатором «Квант -Z» (ООО «Кортекс», Россия), в мкг/л. Сывороточный уровень гормонов (лептин (нг/мл), фолликулостимулирующий и лютеинизирующий гормон (в МЕ/л), тестостерон (нмоль/л)) определялся методом иммуноферментного анализа.

## Статистический анализ

Статистическую обработку полученных данных и их значимость проводили с помощью лицензионного пакета Statistica 6.0, расчет критерия Уилкоксона для независимых выборок с поправкой на множественную проверку гипотез (поправка Бонферрони). Значимость различий определялась при р=0,05.

## Этическая экспертиза

На все проводимые обследования было получено письменное согласие родителей или другого официального представителя и разрешение локального этического комитета. Исследование было одобрено на заседании этического комитета НИИАП РостГМУ от 16 января 2015 г. (протокол №2).

## РЕЗУЛЬТАТЫ

На первом этапе исследования были оценены антропометрические данные и ряд физиологических особенностей организма подростков групп сравнения. Установлено, что у мальчиков-подростков 1-й группы в основном преобладал равномерный тип распределения подкожно-жировой клетчатки (ПЖК) — 59,8%, у 10,5% — гиноидный тип и в 29,7% — андроидный.

Во 2-й группе в 100% распределение ПЖК равномерное. В группах с ожирением отмечался гипергидроз ладоней и стоп (в 1 — 60,0% и 2 — 33,3%) и ложная гинекомастия (в 1 — 59,7% и 2 — 20,8%). SDS ИМТ в 3 группе был достоверно ниже относительно показателей основных групп. Объем яичек в 1 группе был значимо меньше относительно 2 и 3-й групп (таблица 1).

**Table:** Таблица 1. Морфометрическая оценка обследуемых групп Примечание: ИМТ — индекс массы тела; р<0,05 — статистическая значимость различий, р1 — между 1 и 3 группой, р2 — между 2 и 3 группой; р3 — между 1 и 2 группой (с учетом поправки Бонферрони).

Объем тестикул, мл	2,7±0,3	10,6±2,5	13,5±3,1	0,026	0,118	0,003
SDS ИМТ	2,8±0,19	2,1±0,43	0,8±0,10	0,004	0,011	0,470

На втором этапе проводилось гормонально-микроэлементное исследование биологических сред (сыворотка крови и моча).

Оценка особенностей гормонального фона (таблица 2) показала наибольшие значения лептина, на уровне тенденции, у мальчиков-подростков с ожирением в 1-й и 2-й группах и наименьшие — в группе контроля. Уровни гонадотропинов были наименьшими в 1-й группе и наибольшими в 3-й группе. Значимые различия получены с контрольной группой в 1-й группе по уровню ФСГ и ЛГ, а во 2-й — только по уровню ФСГ. Содержание общего тестостерона имело аналогичное распределение с уровнями гонадотропинов. Достоверность отличий установлена относительно контрольной группы в 1-й и 2-й группах.

**Table:** Таблица 2. Гормональный статус мальчиков-подростков групп сравнения Примечание: ФСГ — фолликулостимулирующий гормон, ЛГ — лютеинизирующий гормон, Ts — тестостерон общий; р<0,05 — статистическая значимость различий, р1 — между 1 и 3 группой, р2 — между 2 и 3 группой; р3 — между 1 и 2 группой (с учетом поправки Бонферрони).

Лептин, нг/мл	18,8±3,1	15,9±2,2	3,41±0,35	0,126	0,076	0,333
ФСГ, МЕ/л	1,75±0,16	1,92±0,21	2,87±0,33	0,003	0,027	0,092
ЛГ, МЕ/л	1,18±0,01	2,73±0,18	3,15±0,22	0,011	0,233	0,061
Ts, нмоль/л	7,97±1,25	10,53±1,75	18,1±3,36	0,033	0,005	0,738

При анализе микроэлементного состава мочи у исследуемых мальчиков проведена оценка уровня как ЭХЭ, так и ТХЭ. Из группы ТХЭ изучалась концентрация свинца, а в группе ЭХЭ — цинк, селен, марганец и хром (таблица 3). Содержание свинца было статистически значимо выше в 1-й группе относительно пациентов 2-й группы и группы контроля. Причем у мальчиков с ЗПР абсолютные значения уровней свинца в моче были максимальными. Между тем другая тенденция установлена в содержании ЭХЭ. У мальчиков с ожирением обнаружено достоверно более низкое содержание цинка в сравнении с группой контроля, и минимальные его уровни отмечены в 1-й группе. Другие ЭХЭ тоже были снижены у пациентов с ожирением относительно группы контроля. Статистически значимое снижение селена и хрома выявлено у подростков 1-й группы к группе контроля. У мальчиков основных групп определено более низкое содержание марганца, нежели у подростков с нормальным ИМТ и физиологическими темпами пубертата. Однако статистическая значимость имелась только в группе с ожирением и ЗПР, а во 2-й группе — отличия на уровне тенденции.

**Table:** Таблица 3. Микроэлементный состав мочи мальчиков-подростков групп сравнения Примечание: Рb — свинец, Zn — цинк, Se — селен, Cr — хром, Mn — марганец; р<0,05 — статистическая значимость различий, р1 — между 1 и 3 группами, р2 — между 2 и 3 группами; р3 — между 1 и 2 группами (с учетом поправки Бонферрони).

Pb	11,1±3,2	9,6±0,7	8,2±0,3	0,016	0,44	0,08
Zn	135,7±4,5	202,7±5,3	288,2±6,3	0,015	0,012	0,013
Se	10,6±1,1	15,6±1,3	18,2±1,2	0,016	0,13	0,002
Cr	2,5±0,1	2,8±0,1	3,4±0,2	0,014	0,12	0,50
Mn	2,6±0,3	2,9±0,4	3,4±0,3	0,012	0,21	0,25

## ОБСУЖДЕНИЕ

## Репрезентативность выборок

Набор участников для исследования проводился только в федеральном учреждении.

## Сопоставление с другими публикациями

Механизмы пубертата у девочек и мальчиков различны. Для девочек одним из необходимых триггеров полового созревания считается определенный объем жировой ткани. Ввиду того, что именно в жировой ткани происходит ароматизация андрогенов в эстрогены, так как она является дополнительным пулом эстрогенов на периферии и участником процесса запуска пульс-секреции люлиберина и циклической секреции гонадотропинов в центральной нервной системе. А вот для мальчиков, напротив, избыток жировой ткани и, следовательно, эстрогенов в пубертате ингибирует андрогенные стимулы на органы мишени [3]. Лептин имеет прямую корреляцию с объемом жировых масс в организме человека, и наши данные подтверждают эту зависимость и не только, так как максимальные концентрации лептина в группе с ЗПР являются косвенным подтверждением ретенции лептина на гипоталамо-гипофизарно-гонадную ось и, как следствие, половое развитие [8][9].

Результаты микроэлементной эндосреды у мальчиков установили определенный сдвиг в содержании токсических и эссенциальных МЭ в случае присутствия у подростка ожирения. Вектор смещен в сторону преобладания уровней ТХЭ, в частности свинца, и снижения ЭХЭ — цинк, селен, хром и марганец. Причем у мальчиков с ЗПР наблюдались максимальные концентрации свинца в моче. По нашему мнению, этому может быть несколько возможных объяснений. Прежде всего, результат техногенного загрязнения атмосферы способствует высокому содержанию этого ТХЭ в организме детей, проживающих на загрязненной территории. В основном кумуляция свинца происходит в жировой ткани, которая как «щит», защищая другие органы и ткани от негативного влияния тяжелых металлов, адсорбирует их в себе, соответственно, уровни свинца будут преобладать у детей с ее избытком [10][14]. Еще в литературе описываются свойства свинца напрямую стимулировать гены печеночного глюконеогенеза и в высоких концентрациях вызывать нарушение углеводного обмена, вплоть до сахарного диабета 2 типа, и дислипидемию, способствуя формированию ожирения [15][16]. Помимо прочего, для ожирения характерен повышенный оксидативный стресс, и детский организм не является исключением, а состояние тканевой гипоксии способствует нарушению перфузии и кумуляции тяжелых металлов в тканях и клетках организма. Избыточные концентрации свинца в организме ребенка, страдающего ожирением, в период полового созревания крайне негативно сказываются на синхронности работы звеньев гипоталамо-гипофизарно-тестикулярной оси, так как свинец обладает прямым и опосредованным повреждающим действием на различные звенья этой функциональной системы [17], и это подтверждают результаты нашего исследования. В группе детей с ожирением и без вторичных половых признаков определены значимо низкие концентрации тестостерона и гонадотропинов в отличие от групп сравнения, где имело место нормальное становление вторичных половых признаков.

Рассуждая о цинке, известно его положительное влияние на репродуктивную систему. Он обладает способностью накапливаться в половых органах и стимулировать развитие вторичных половых признаков. В связи с чем дефицит этого ЭХЭ в период полового созревания может приводить к задержке пубертата. Зная о влиянии низких концентраций цинка на формирование лептинорезистентности и гиперлептинемии [18], наши результаты согласуются и подтверждают роль дефицита цинка в процессах девиации метаболизма и прогрессии ожирения у мальчиков-подростков.

Как и цинк, селен ответственен за развитие функционального слоя мужских половых гонад, защиту сперматозоидов от окислительного стресса, в связи с чем становится понятно, почему у мальчиков-подростков с ЗПР уровень данного ЭХЭ снижен [13]. Olechnowicz J. с соавт. (2017 г.) в своем исследовании тоже показали, что концентрация селена снижена у пациентов с ожирением, независимо от возраста и пола [19], и имеет обратную связь с инсулинорезистентностью.

Если же говорить о влиянии хрома на репродуктивную ось и метаболизм, то указывается, что его дефицит, который наблюдался у мальчиков-подростков с ожирением и в нашем исследовании, способствует нарушению репродуктивной функции у мужчин, толерантности к глюкозе, инсулинорезистентности и ожирению. С другой стороны, употребление высокоуглеводистой пищи приводит к снижению уровня хрома. То есть формируется «замкнутый круг»: инсулинорезистентность и гиперинсулинизм, нарушение толерантности к глюкозе, при недостатке хрома, которые вызывают изменение пищевого поведения по типу переедания и, как следствие, сбой липогенеза и ожирение, что еще больше усугубляет дефицит этого МЭ, так как препятствует накоплению хрома в организме [20].

Марганец — важный элемент, участвующий в синтезе и активации многих ферментов, а также в регуляции метаболизма глюкозы и липидов у людей. Он один из необходимых компонентов супероксиддисмутазы, отвечающей в основном за улавливание активных форм кислорода при окислительном стрессе митохондрий. И как дефицит, так и интоксикация Mn связаны с неблагоприятными метаболическими и психоневрологическими эффектами. Mn всасывается через желудочно-кишечный тракт и быстро концентрируется в органах, богатых митохондриями, такими как печень, поджелудочная железа и гипофиз [10][11]. Такой вариант микроэлементоза у мальчиков-подростков с ожирением, возможно, указывает на присутствие в их организме избытка продуктов окисления на фоне тормозящих влияний в центральных отделах репродуктивной оси, что в случае более выраженного дефицита Мn, который установлен нами в 1 группе, предрасполагает к ЗПР.

Суммируя полученные результаты и известные мнения о гормонально-микроэлементной изменчивости в организме мальчиков-подростков в период пубертата, установленные особенности уровня лептина, как важного коррелята липидного и углеводного обмена [2][9], и определенное соотношение ряда токсических и эссенциальных микроэлементов может внести определенный вклад в патогенез задержки полового развития у мальчиков с ожирением. Возникающая диссоциация микроэлементов в результате экзо- и эндогенных воздействий, по-видимому, может вызвать нарушение метаболизма, сдвиги углеводного и липидного обмена, тем самым активируя лептин и резистентность рецепторной системы к нему, избыточные количества свободных фракций которого, в свою очередь, отрицательно сказываются на гомеостазе микроэлементов [10][14].

## Клиническая значимость результатов

Таким образом, определение уровня лептина и ряда химических микроэлементов, таких как свинец, цинк, селен, хром и марганец, может использоваться в качестве прогностической и диагностической панели возможного отклонения пубертата у мальчиков-подростков с КЭО. В случае же имеющейся ЗПР определение этих биологических маркеров позволит наиболее оптимально определить лечебную стратегию, максимально эффективно восстановить морфофункциональный сдвиг и минимизировать риски репродуктивных расстройств для здоровья в дальнейшем.

## Ограничения исследования

Как таковых явных ограничений в проведенном исследовании нет. Однако, возможно, могут быть какие-то смещения результатов. Нами не брался в учет национальный или расовый фактор. Не стоит исключать, что исследование проведено на небольшой выборке мальчиков-подростков в одном учреждении, то есть его следует отнести к одноцентровому пилотному исследованию с оценкой «случай-контроль». Ну и, конечно же, не проводился сравнительный межтерриториальный анализ. Исследованная группа подростков проживает в пределах Ростовской области, в одной экологической нише, имеющей определенную схожую долю распределения микроэлементов в окружающей среде.

## Направления дальнейших исследований

Мы планируем продолжить эту работу по изучению особенностей и влияния на метаболические процессы и половое развитие мальчиков-подростков гормонально-микроэлементного состава внутренней биологической среды с учетом устранения перечисленных факторов, которые не были введены в данное пилотное исследование.

## ЗАКЛЮЧЕНИЕ

Результаты нашего исследования показали, что в организме мальчиков подростков с ожирением и ЗПР имеется избыток лептина, накопление тяжелых металлов и дефицит эссенциальных микроэлементов. Полагаем, что такой биохимический фон будет способствовать прогрессированию окислительного стресса, приводя к тканевой гипоксии, нарушая функциональную активность репродуктивной оси, на что указывает низкое содержание тестостерона и гонадотропных гормонов. Менее выраженные сдвиги в уровне лептина и микроэлементов у мальчиков-подростков с ожирением, но без признаков ЗПР, определяет сбой в нейроэндокринной регуляции, и не оказывает влияние на половое развитие. Напротив, гомеостаз гормонально-микроэлементного состава биологических сред организма мальчиков контрольной группы без отклонений в метаболизме и половом созревании обеспечивает гармоничное их развитие. Следовательно, микроэлементный дисбаланс у мальчиков-подростков с КЭО является очень важным и одним из решающих факторов, стимулирующих задержку полового развития.

## ДОПОЛНИТЕЛЬНАЯ ИНФОРМАЦИЯ

Источники финансирования. Исследование проведено в рамках выполнения государственного задания.

Конфликт интересов. Авторы декларируют отсутствие явных и потенциальных конфликтов интересов, связанных с содержанием настоящей статьи.

Участие авторов. Все авторы одобрили финальную версию статьи перед публикацией, выразили согласие нести ответственность за все аспекты работы, подразумевающую надлежащее изучение и решение вопросов, связанных с точностью или добросовестностью любой части работы.

Благодарности. Коллектив авторов выражает благодарность Дышековой Оксане Викторовне за помощь в переводе оригинальных статей на английском и немецком языках, заведующей отделением клинико-диагностической лаборатории НИИАП РостГМУ за помощь в проведении лабораторных исследований.
